# Effect of Probability Information on Bayesian Reasoning: A Study of Event-Related Potentials

**DOI:** 10.3389/fpsyg.2019.01106

**Published:** 2019-05-14

**Authors:** Zifu Shi, Lin Yin, Jian Dong, Xiang Ma, Bo Li

**Affiliations:** Cognition and Human Behavior Key Laboratory of Hunan Province, School of Educational Science, Hunan Normal University, Changsha, China

**Keywords:** Bayesian reasoning, base rate, hit rate, ERPs, “anchoring-adjustment” heuristic

## Abstract

People often confront Bayesian reasoning problems and make decisions under uncertainty in daily life. However, the time course of Bayesian reasoning remains unclear. In particular, whether and how probabilistic information is involved in Bayesian reasoning is controversial, and its neural mechanisms have rarely been explored. In the current study, event-related potentials (ERP) were recorded from 18 undergraduates who completed four kinds of Bayesian reasoning tasks. It was found that compared with the high hit rate task, the low hit rate task elicited more significant N1 (100∼200 ms) and N300 (250∼350 ms) components, suggesting that N1 might be associated with the attention to stimulus materials, and N300 might be associated with the anchor to hit rate. In contrast to the low base rate task, the high base rate task elicited more significant late positive components (LPC, 350∼700 ms), indicating that LPC might reflect the adjustment of probability estimation based on the base rate. These results demonstrate that both the base rate and hit rate play significant roles in Bayesian reasoning, and to some extent, these findings verify that people may follow the “anchoring-adjustment” heuristic in Bayesian reasoning. The current findings provide further proof for the information processing mechanism of Bayesian reasoning.

## Introduction

People are prone to adjust the existing point of view according to new emerging information or evidence to make an appropriate judgment and decision under conditions of uncertainty, called Bayesian inference (Samuel and Wu, 2000). Although Bayesian reasoning is vital in our daily lives, the performance by an individual is poor. The Bayesian reasoning problem textual paradigm (Gigerenzer and Hoffrage, 1995) is as follows:

The probability of breast cancer in the population is 1% for a woman who participates in a routine screening. If a woman has breast cancer, the probability that she will have a positive mammography is 80%. If a woman does not have breast cancer, the probability that she will also have a positive mammography is 9.5%. If a woman in this group had a positive mammography, what’s the probability that she has breast cancer?

The probability information can be written according to Bayes’ rules:

$$\text{P}(\text{d}|\text{h})=\frac{\text{P}(\text{h})\text{P}(\text{d}|\text{h})}{\text{P}(\text{h})\text{P}(\text{d}|\text{h})+\text{P}(-\text{h})P(\text{d}|-\text{h})}$$

In the equation above, P(h) stand for the base rate as 1%, P(d|h) represents the hit rate as 80%, P(d|−h) is the false alarm rate as 9.6%, and P(h|d) is the posterior probability. Whether people follow the Bayesian rules in the Bayesian reasoning process has been widely debated since Edward initially researched the Bayesian reasoning process in the 1960s (Edwards, 1968; Slovic et al., 1976; Gould, 1992; Tang and Shi, 2011). Tversky and Kahneman (1974) introduced the concept of “base rate neglected” to explain the human reasoning process and they argued that people ignored the base rate 1% when did the Bayesian reasoning problems then made an overestimate result. Samuel and Wu (2000) suggested that people didn’t follow the Bayesian rules when did calculation in the reasoning process and they proposed “to assume a probability value – to find the evidence – to modify the probability value,” or “anchoring and adjusting” strategy for short, might be the main strategy of human reasoning (Shi and Zhang, 2009). However, whether probabilistic information is fully involved in Bayesian reasoning and whether its calculations follow Bayesian criteria are not supported by empirical evidence.

As a kind of classical probabilistic reasoning, Bayesian reasoning consists of the selection and processing of various probabilistic, and arithmetic information. However, the time course of Bayesian reasoning in the context task is still unclear, whether the base rate and hit rate were used in Bayesian reasoning and whether the calculation of probability information follows Bayesian rules was deduced reversely, and indirectly on the basis of reasoning results for the subjects in existing studies. Researchers found that the fixation time of the probability information can reflect the attention and use of the information and confirmed the possibility to speculate on the reasoning process of the subjects (Cohen and Staub, 2015; Reani et al., 2017). An eye movement study that monitored and analyzed the time course in the reasoning process revealed that people did not neglect the base rate and presented three stages of the Bayesian reasoning process. Stage 1 is the problem representation stage, in which attention to stimulus materials is involved in the reasoning process; Stage 2 consists of information integration and the selection of probability estimation strategy, in which people construct and select the “anchoring and adjusting” probability estimation strategy; and Stage 3 is the probability judgment stage (Shi et al., 2015).

Event-related potential (ERP) techniques have been applied to observe the time course of higher-level cognitive functions in many other studies, such as reasoning, problem solving, etc., as these techniques can provide a visual indicator for information processing (Liang et al., 2010). Some related ERP components were identified and discussed. Chen et al. (2005) adopted ERP to explore the neural mechanism of inductive reasoning and found that the amplitude of the late positive component (LPC) for the inductive task was significantly higher; namely, subjects consumed more cognitive resources and mental energy when conducting inductive tasks. Similarly, the LPC and N2 (200 ∼ 300 ms) amplitudes were also found to be positively correlated with the input amount of psychological resources in series Bayesian reasoning studies (Kolossa et al., 2015; Kopp et al., 2016; Seer et al., 2016). An urn-ball task was used to investigate the neural bases of the cognitive processes of Bayesian reasoning. The LPC (P3a, P3b, and slow wave) provided dissociable measures of the Bayesian reasoning process, and the N2 (N300) component was stronger when the prior probability could not be computed (Seer et al., 2016). This research did not address the N2 wave and attributed the prior probability to the P3a wave.

In many other studies, the N2 (N300) components were related to the anchoring effect. The prior probability cannot be computed means the anchoring effect needs more cognitive resources and arouses stronger N300. The study of Qu et al. (2008) provided strong evidence for the anchoring effect related to N300, and the N300 and LPC were aroused by psychological scale and reflected the same psychological component – psychological calculation, thus supporting the anchor adjustment heuristic model. Additionally, N300 was detected to be a representation of algorithmic feature recognition and selection (Zhou et al., 2006). However, Kolossa et al. (2015) provided an example for us to combine the ERP techniques and the context Bayesian reasoning paradigm in this study. In this way, the time procedure of the Bayesian reasoning process was explored, and whether and how probabilistic information is involved in Bayesian reasoning would be tested.

Therefore, this study assumes that: (1) People do not neglect the base rate; thus, both the base rate and hit rate information plays an important role in the Bayesian inference process. (2) The calculation of probability information does not follow the Bayesian rules, and the “anchoring – adjustment” heuristic strategy is adopted in Bayesian inference.

## Experiment

### Purpose and Hypothesis

The experiment aimed to research how base rate and hit rate effect on the Bayesian reasoning textual paradigm. This study hypothesized that the EEG data shows a significant difference in the amplitude and latency of ERP components induced by high and low level basal rate and hit rate task. (2) The calculation of probability information does not follow the Bayesian rules, and the “anchoring – adjustment” heuristic strategy is adopted in Bayesian inference; therefore, the EEG data showed N300 related to the anchoring effect, whereas the LPC components related to the adjustment procedure.

### Materials and Methods

#### Participants

This experiment was approved by the Ethics Committee of Hunan Normal University in China, and written informed consent was obtained from all participants prior to the experiment. Twenty paid students were recruited from a university for this study, ten males and ten females. The average age of the subjects was 20.55 years, ranging from 19 to 22 years. All participants were right-handed and had no physical or mental illness and had normal eyesight and keyboard operation ability. Two students were removed for showing relatively higher artifacts. Thus, eight males and ten females, whose average age was 20.38 years, were recorded validly.

#### Materials

**FIGURE 1:**
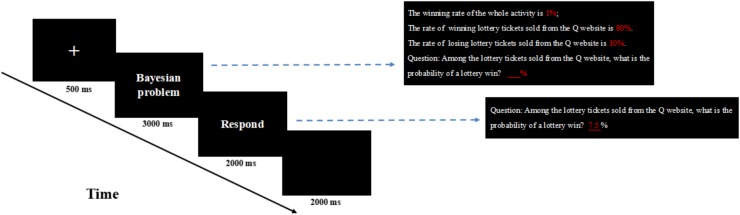
The presentation of the Bayesian reasoning stimulates.

This experiment used the classical Bayesian reasoning problem, with two levels of base rate and hit rate, high and low, and a false positive of 10%. The participants needed to make posterior probability estimations based on the probabilistic information given in the problems. Thus, there were four conditions: low base rate and low hit rate (low-low for short), low base rate and high hit rate (low-high for short), high base rate and low hit rate (high-low for short), and high base rate and high hit rate (high-high for short) (Tang and Shi, 2011). In addition, to strictly control the irrelevant variables and meet the needs of the ERP experiment, the experimental materials were reorganized as follows: an integer was chosen randomly as the base rate and hit rate from 70 to 80% (high level) and 1–10% (low level) to construct 100 reasoning problems of each kind, i.e., “low-low,” “low-high,” “high-low,” “high-high,” and “high-high.” Then, 35 Bayesian reasoning problems were chosen as formal experimental materials for each kind, namely, 140 problems were chosen in total. For example, the “low-high” experiment could be constructed when 1% was chosen as the low-level base rate and 80% as the high-level hit rate, and the probabilistic information in the problems was marked in red to for easier visualization and comprehension in reading (see Figure 1).

#### Procedure

The participants were seated 60 cm away from a computer screen and tasked to estimate the Bayesian problems. Some practices were made prior to the experiment to ensure that the participants could understand and become familiar with the experimental task.

The formal experiment consisted of 2 blocks, and there were 70 questions for each block. The questions were presented in a random order in each block, and the blocks were counterbalanced between participants.

The procedure is illustrated in Figure 1. A “+” was shown in the center of the screen at the beginning of a trial for 500 ms, and the Bayesian problem appeared on the screen. The subjects were asked to press the space key within 3000 ms to enter the answer interface and then press the estimated value relying on the integer in 2000 ms. Finally, a 2000 ms empty screen was shown before the next trial began. The tasks were presented in the style of black background and white foreground, except for the two numbers highlighted in red. After each block, the participants were permitted to take a rest for 2 min.

#### EEG Recording

EEG was collected by 10∼20 system expansion 64-channel electrode caps produced by Brain Products of Germany. The two mastoids in the ears were linked and served as the reference electrodes. Two channels were placed at the outside canthi and downside canthi of oculus dexter to record the horizontal electrooculogram (HEOG) and vertical electrooculogram (VEOG). The impedance of all electrodes was maintained below 5 kΩ. The bandpass of the filtering was set at DC∼100 Hz. The sampling rate was 500 Hz.

#### ERP Data Analyses and Statistics

After recording the EEG, the offline method was used to analyze the datum. Then, trials with amplitudes over ± 80 μv, after autocorrecting VEOG and HEOG, were observed as artifacts, and removed. The Bayesian reasoning process occurred after the tasks were presented; thus, an epoch from 200 ms prestimulus until 800 ms poststimulus was chosen for analysis, and 200 ms prestimulus served as the baseline. According to the kinds of Bayesian reasoning tasks, the EEG needed to be overlaid and averaged, and trials with artifacts or unfinished tasks were removed. The overlaid times for each kind of task exceeded 30 trails. Based on this study, the total average chart and the voltage topographic map, nine electrode points located in the central part of the brain were selected (FC_Z_, FC1, FC2, C_Z_, C1, C2, CP_Z_, CP1, and CP2) (Kolossa et al., 2015), and a three factors repeated measure variance analysis (ANOVA) of each electrode point was conducted. Three factors included the base rate, the hit rate and the recording spot. The *P* value of variance analysis was revised by the Greenhouse – Geisser method, and the EEG topographic map was drawn based on data from the 64 channels.

**Table 1:** The average posterior probability and reaction times for the four reasoning tasks from 18 participants (M ± SD).

Task type	Probability estimates	Response time (ms)
Low base rate – low hit rate	14.92 ± 15.00	1072.21 ± 306.07
Low base rate – high hit rate	35.99 ± 24.60	1162.55 ± 401.19
High base rate – low hit rate	32.69 ± 19.60	1168.17 ± 392.50
High base rate – high hit rate	66.13 ± 14.22	1112.32 ± 371.24

## Results

### Behavioral Results

In this study, the posterior probability was estimated, and the reaction times for four reasoning tasks are given in Table 1.

By repeated measures ANOVA of the reaction time, the results indicated that there was no significant main effect of the hit rate and the base rate [*F*(1,17) = 0.54, *p* > 0.05, η_p_^2^ = 0.03; *F*(1,17) = 0.36, *p* > 0.05, η_p_^2^ = 0.02], and the effect between the base rate and hit rate was significant [*F*(1,17) = 7.25, *p* < 0.05, η_p_^2^ = 0.30]. Furthermore, the reaction time in the low-low conditions was significantly less than that in the low-high conditions. The simple effect test showed that the simple effect of the hit rate was significant in the low level base rate condition [*F*(1,17) = 7.02, *p* < 0.05]. The reaction time in the high hit rate task was longer than that in the low hit rate task. The simple effect of the hit rate was not significant in the high level base rate condition [*F*(1,17) = 1.60, *p* > 0.05]. This finding indicates that the effect of the hit rate was affected by the low base rate.

### EEG Results

**FIGURE 2:**
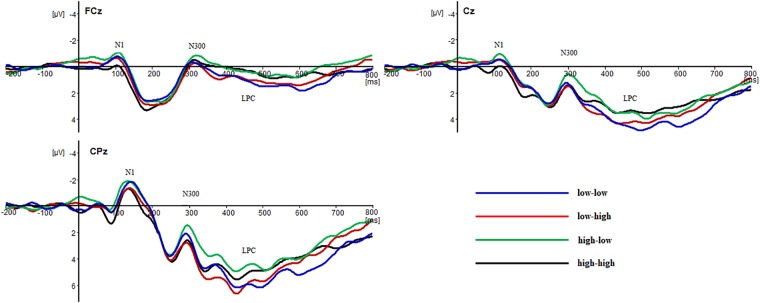
The grand average of event-related potentials (ERP) elicited for the four Bayesian reasoning tasks.

The ERP results induced by the four reasoning tasks are shown in Figure 2.

**FIGURE 3:**
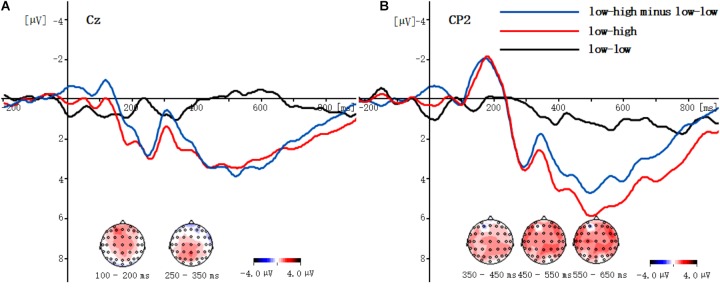
The difference waves in Cz **(A)** and CP2 **(B)** (“low-high” task minus “low-low” task) and the voltage topographic maps.

According to the grand mean (see Figure 2) and the differential wave topographic map (see Figure 3) in this study, 100∼200 ms (N1), 250∼350 ms (N2), and 350∼700 ms (LPC) were identified as time windows for the ERP components analysis, and ANOVA was used to analyze the base rate, hit rate and electrode point. The results were as follows.

### The Average Amplitude of the Time Window: 100∼200 ms (N1)

Three factors repeated measures ANOVA of the average amplitude of 100∼200 ms found that the main effect of the hit rate was significant, and the elicited amplitude in the low hit rate task was more negative than that in the high hit rate task [*F*(1,17) = 13.89, *p* < 0.01, η_p_^2^ = 0.45]. In addition, the main effect of the electrode point was significant, CPz > FCz > Cz [*F*(8,136) = 15.30, *p* < 0.01, η_p_^2^ = 0.47]. The main effect of the base rate was not significant [*F*(1,17) = 0.34, *p* > 0.05, η_p_^2^ = 0.02], and the interaction of the base rate and hit rate was not significant [*F*(1,17) = 0.87, *p* > 0.05, η_p_^2^ = 0.05]. In addition, there were no significant interaction effects between the electrode point and the base rate [*F*(8,136) = 0.45, *p* > 0.05, η_p_^2^ = 0.03] or between the electrode point and the hit rate [*F*(8,136) = 0.55, *p* > 0.05, η_p_^2^ = 0.03].

### The Average Amplitude of the Time Window: 250∼350 ms (N2)

Three factors repeated measures ANOVA of the average amplitude of 250 350 ms demonstrated a significant main effect of the hit rate, and the elicited amplitude in the low hit rate task was more negative than that in the high hit rate task [*F*(1,17) = 7.92, *p* < 0.05, η_p_^2^ = 0.32]. In addition, the main effect of the electrode point was significant, CPz > FCz > Cz, [*F*(8,136) = 10.74, *p* < 0.01, η_p_^2^ = 0.39]. However, no significant main effect was observed for the base rate [*F*(1,17) = 2.59, *p* > 0.05, η_p_^2^ = 0.13], no significant interaction was observed for the base rate, and the hit rate was not significant [*F*(1,17) = 0.07, *p* > 0.05, η_p_^2^ = 0.01]. In addition, the interaction of the electrode point and the base rate was not significant [*F*(8,136) = 0.57, *p* > 0.05, η_p_^2^ = 0.03], and the interaction of the electrode point and the hit rate was not significant [*F*(8,136) = 1.53, *p* > 0.05, η_p_^2^ = 0.08].

### The Average Amplitude of Time Window: 350∼700 ms (LPC)

Three-factor repeated measures ANOVA of the average amplitude of 350∼700 ms indicated that the main effect of the base rate was significant, and the elicited amplitude in the high hit rate task was more positive than that in the low hit rate task [*F*(1,17) = 8.77, *p* < 0.01, η_p_^2^ = 0.34]. The main effect of the electrode point was significant, CPz > FCz > Cz [*F*(8,136) = 21.45, *p* < 0.01, η_p_^2^ = 0.56], while the main effect of the hit rate was not significant [*F*(1,17) = 0.13, *p* > 0.05, η_p_^2^ = 0.01], and there were no significant interaction effect between the base rate and hit rate [*F*(1,17) = 0.12, *p* > 0.05, η_p_^2^ = 0.01]. In addition, the interaction of the electrode point and the base rate [*F*(8,136) = 0.90, *p* > 0.05, η_p_^2^ = 0.05] and the interaction of the electrode point and the hit rate were not significant [*F*(8,136) = 0.78, *p* > 0.05, η_p_^2^ = 0.04].

### The Different Wave and Topographic Maps in Different Hit Rate Tasks

Considering the grand mean (see Figure 2) and the analyses of the behavioral results, we could see that the effect of the hit rate was affected by the low base rate. Thus, the performance of the subjects in “low-low” and “low-high” tasks was selected to conduct the different wave analysis.

Figure 3A shows that compared to the high hit rate task in the 100∼200 ms time window, the low hit rate task could elicit a more negative component at Cz.

Figure 3B indicates that compared to the low base rate task in the 350∼700 ms time window, the high base rate task could elicit a larger positive component, and the different waves manifested as the LPC at CPz.

**FIGURE 4:**
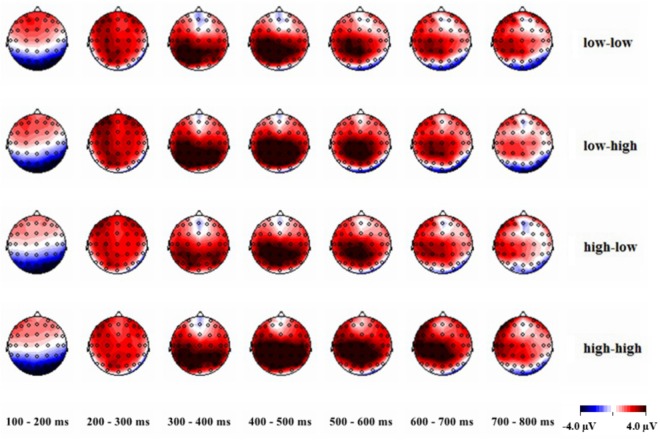
Comparison of the voltage topographic maps for the four different tasks.

Analysis of the topographic maps for the 4 types of tasks every 100 ms. As shown in Figure 4, four different types of reasoning tasks activate the temporal lobe, the parietal occipital region and the lateral region of the frontal. Combined with the different wave topographic map in Figure 3, it can be further concluded that, compared with the high hit rate task, the low hit rate task mainly activates the prefrontal and the parietal regions. Compared with the low-rate task, the high-rate task activates parts of the parietal, lobi temporalis and frontal lobes.

## Discussion

### The Role of the Base Rate and Hit Rate in Bayesian Inference

To a certain extent, this study provided new evidence for the fact that both the base rate and hit rate played important roles in Bayesian inference. First, high and low base rate information had a significant influence on the posterior probability estimation, and there were significant differences in amplitude induced by high and low base rate information in LPC. This result showed that the participants pay attention to the base rate information in Bayesian reasoning. Second, in the 100∼200 ms time window, the low hit rate task evoked a more negative component than the high hit rate task, indicating that the brain processing stages underlying the high, and low hit rates were different. Third, in the 250∼350 ms time window, the low hit rate task evoked a more negative component than the high hit rate, and the N300 peak appeared; then, LPC appeared in the next window. This finding might suggest that in Bayesian reasoning, the subjects initially anchored the hit rate information, adjusted up and down in line with the base rate, and then finally obtained the estimated posterior probability value. In this way, the hit rate information was successful at this stage. The current results also showed that the hit rate information was processed earlier than the base rate information in the time course. These results were completely consistent with the results of a previous eye movement experiment (Shi et al., 2015) of Bayesian reasoning, namely, the subjects paid more attention to the hit rate than the base rate, indicating that hit rate got processed at a priority. The present study has provided further evidence, different from the eye movement results, from the proposed model of Bayesian reasoning and in accordance with Kopp et al. (2016), suggesting that probability information played an important role in the reasoning task, even in the Bayesian inference textual paradigm, and shows a significant difference in N300 and LPC between the high and low level of the base rate and hit rate.

### The “Anchoring and Adjustment” Heuristic in Bayesian Reasoning

Tversky and Kahneman (1974) introduced the concept of the “heuristic method” and used it to explain why the human mind does not conform to the rules of logic. Samuel and Wu (2000) proposed that people may employ the “anchor and adjustment” strategy to conduct Bayesian reasoning. Subsequently, some attempts have been made to verify the proposed strategy/theory for Bayesian reasoning (Zhu and Gigerenzer, 2006; Shi and Zhang, 2009). However, most of these studies mainly conjectured the reasoning process only from the posterior probability and cannot fully explain the “base rate neglected” effect as well as how people reasoned by using the “anchoring and adjustment” heuristic.

Based on previous studies, this study further used the ERP technique to directly examine the Bayesian reasoning process. First, the behavior performance indicates that both the hit rate and the based rate are considered simultaneously during the Bayesian reasoning process, as the two main effects of the base rate and hit rate were significant, and the posterior probability varied with the level of the two kinds of probability information. These results supported the view that “the participants do not ignore the base rate” (Over and Perham, 2002; Shi and Zhang, 2009; Johnson and Tubau, 2015). This idea is also consistent with that of previous studies (Tang and Shi, 2011). Second, the ERP results have provided direct evidence (at least partially) for the “anchoring and adjustment” strategy, as the base rate and hit rate evoked N300 related to the anchoring based on accessibility, and LPC was associated with the anchoring based on adjustment (Qu et al., 2008; Kolossa et al., 2015). This finding indicates that people may adopt an “anchorage-adjustment” strategy in the Bayesian reasoning process, but which components accurately link to the anchoring effect and adjustment effect also need further research.

### Three-Stage Model of the Bayesian Reasoning Process

This study provided electrophysiological evidence for the time course of probability information processing in Bayesian reasoning. In the 100∼200 ms time window, compared with the high hit rate task, the low hit rate task elicited a more negative N1 in the frontal region. The N1 components appeared after the stimulation was presented, and was significantly affected by attention, which showed the increase of amplitude. It’s related to the visual auditory stimulation and attention. This earlier ERP component might reflect the early visual processing of stimuli in Bayesian reasoning. This result was congruent with a previous study indicating that N1 was associated with the attention to stimulus materials in the reasoning process (Lei et al., 2010). Kolossa et al. (2015) and Seer et al. (2016) used urn-ball task to investigate the neural bases of Bayesian inference. They argued that the N2 and P3 comonents has been considered to anticipate events and to react to unexpected discrepancies (Hillyard and Picton, 1987). What’s more, their researches found that the N2 amplitudes were enhanced when probability informations were unknown. Then, in the 250∼350 ms time window, the difference of N300 elicited by the high or low hit rate information was significant in our research, which might suggest that N300 in this study was associated with the anchor to the hit rate. This explanation is also consistent with the anchoring effect from Qu et al. (2008). In the 350∼700 ms time window, the high base rate task elicited a more significant LPC compared with the low base rate task. According to the current experimental task, it was argued that LPC might be correlated with the adjustment of the probability estimation based on the base rate.

Together, the process of Bayesian reasoning could be divided into three stages: first, Bayesian reasoning tasks were visually processed as reflected by N1. Second, the “anchoring and adjusting” heuristic method was used to solve Bayesian reasoning tasks as reflected by N300 and LPC. Third, the posterior probability was estimated. This idea is consistent with the results from Shi et al. (2015), which suggested the three-stage model by using eye movement technique, we may research this theory next.

In summary, the neural bases and time process in Bayesian reasoning were investigated by combining the classical Bayesian reasoning paradigm with ERPs in this experiment. The present results demonstrated that people did not neglect the base rate in Bayesian inference, and both the base rate and the rate had an important effect on the Bayesian reasoning process. The calculation of probability information did not follow the Bayesian rules, while the “anchoring – adjustment” heuristic strategy was adopted in Bayesian inference. These findings have new implications for an in-depth understanding of the time procedure of Bayesian reasoning as well as the functional role of probability information in the reasoning process.

## Ethics Statement

This experiment was approved by the Ethics Committee of Hunan Normal University in China, and written informed consent was obtained from all participants prior to the experiment.

## Author Contributions

ZS, LY, and JD contributed to conception and design of the study and wrote the first draft of the manuscript. JD, XM, and BL performed the experimental procedures and organized the participants for the experiments. LY and JD analyzed the data. XM and BL wrote sections of the manuscript. All authors contributed to manuscript revision, read, and approved the submitted version.

## Conflict of Interest Statement

The authors declare that the research was conducted in the absence of any commercial or financial relationships that could be construed as a potential conflict of interest.
