# Corrigendum: Effect of Probability Information on Bayesian Reasoning: A Study of Event-Related Potentials

**DOI:** 10.3389/fpsyg.2019.02182

**Published:** 2019-09-24

**Authors:** Zifu Shi, Lin Yin, Jian Dong, Xiang Ma, Bo Li

**Affiliations:** Cognition and Human Behavior Key Laboratory of Hunan Province, School of Educational Science, Hunan Normal University, Changsha, China

**Keywords:** Bayesian reasoning, base rate, hit rate, ERPs, “anchoring-adjustment” heuristic

In the published article, there was an error in affiliations 1 and 2. Instead of “Cognition and Human Behavior Key Laboratory of Hunan Province, Changsha, China,” and “School of Educational Science, Hunan Normal University, Changsha, China,” it should only be “Cognition and Human Behavior Key Laboratory of Hunan Province, School of Educational Science, Hunan Normal University, Changsha, China.”

The authors apologize for this error and state that this does not change the scientific conclusions of the article in any way. The original article has been updated.

